# Supplementary material for: Carpenter bee thorax vibration and force generation inform pollen release mechanisms during floral buzzing
Source: Sci Rep. 2022 Aug 5;12:12654. doi: 10.1038/s41598-022-16859-z (PMC9355986; doi:10.1038/s41598-022-16859-z)
Supplement: Supplementary file 2 — Supplementary Video Legend. [file 41598_2022_16859_MOESM2_ESM.docx]

Video showing a carpenter bee defensively buzzing while mounted to a 6-axis force-torque transducer.
